# Increased oxidative stress and apoptosis in the hypothalamus of diabetic male mice in the insulin receptor substrate-2 knockout model

**DOI:** 10.1242/dmm.023515

**Published:** 2016-05-01

**Authors:** Eva Baquedano, Emma Burgos-Ramos, Sandra Canelles, Agueda González-Rodríguez, Julie A. Chowen, Jesús Argente, Vicente Barrios, Angela M. Valverde, Laura M. Frago

**Affiliations:** 1 Department of Paediatrics, Universidad Autónoma de Madrid, Av. Menéndez Pelayo, 65, Madrid 28009, Spain; 2 Department of Endocrinology, Hospital Infantil Universitario Niño Jesús, Av. Menéndez Pelayo, 65, Madrid 28009, Spain; 3 Instituto de Investigación Sanitaria Princesa, IIS-IP, Madrid E-28006, Spain; 4 Centro de Investigación Biomédica en Red de Fisiopatología de la Obesidad y Nutrición (CIBEROBN), Instituto de Salud Carlos III, Madrid E-28029, Spain; 5 Instituto de Investigaciones Biomédicas Alberto Sols, Consejo Superior de Investigaciones Científicas/Universidad Autónoma de Madrid, Madrid E-28029, Spain; 6 Centro de Investigación Biomédica en Red de Diabetes y Enfermedades Metabólicas Asociadas (CIBERDEM), Instituto de Salud Carlos III, Madrid E-28029, Spain

**Keywords:** Diabetes, Cell death, IRS2, Apoptosis, Oxidative stress, Hypothalamus

## Abstract

Insulin receptor substrate-2-deficient (*IRS2*^−/−^) mice are considered a good model to study the development of diabetes because IRS proteins mediate the pleiotropic effects of insulin-like growth factor-I (IGF-I) and insulin on metabolism, mitogenesis and cell survival. The hypothalamus might play a key role in the early onset of diabetes, owing to its involvement in the control of glucose homeostasis and energy balance. Because some inflammatory markers are elevated in the hypothalamus of diabetic *IRS2*^−/−^ mice, our aim was to analyze whether the diabetes associated with the absence of IRS2 results in hypothalamic injury and to analyze the intracellular mechanisms involved. Only diabetic *IRS2*^−/−^ mice showed increased cell death and activation of caspase-8 and -3 in the hypothalamus. Regulators of apoptosis such as FADD, Bcl-2, Bcl-xL and p53 were also increased, whereas p-IκB and c-FLIP_L_ were decreased. This was accompanied by increased levels of Nox-4 and catalase, enzymes involved in oxidative stress. In summary, the hypothalamus of diabetic *IRS2*^−/−^ mice showed an increase in oxidative stress and inflammatory markers that finally resulted in cell death via substantial activation of the extrinsic apoptotic pathway. Conversely, non-diabetic *IRS2*^−/−^ mice did not show cell death in the hypothalamus, possibly owing to an increase in the levels of circulating IGF-I and in the enhanced hypothalamic IGF-IR phosphorylation that would lead to the stimulation of survival pathways. In conclusion, diabetes in IRS2-deficient male mice is associated with increased oxidative stress and apoptosis in the hypothalamus.

## Abstract (precis)

**Summary:** Only IRS2-deficient male mice that become diabetic show apoptosis in the hypothalamus and this is associated with an increase in oxidative stress and inflammatory signals. Controlling inflammation and oxidative stress could prevent diabetes-induced brain injury.

## INTRODUCTION

Insulin receptor substrates (IRSs) mediate the pleiotropic effects of insulin-like growth factor-I (IGF-I) and insulin on metabolism, mitogenesis and cell survival. Upon activation of receptors for insulin or IGF-I, phosphorylation of several IRSs occurs, leading to the activation of major regulatory intracellular pathways involved in proliferation and metabolism (White, 2014). Male *IRS2*-deficient mice (*IRS2*^−/−^) are used as a type 2 diabetes model because they exhibit defects in hepatic insulin signalling, resulting in impaired suppression of glucose production (Withers et al., 1998; Kubota et al., 2000; Previs et al., 2000), and β-cell failure due to disruption of the IGF-I receptor (IGF-IR) mitogenic signalling (Withers et al., 1999) and increased β-cell apoptosis (Lingohr et al., 2003). Furthermore, IRS2-deficient mice recapitulate the fulminate onset of human diabetes because a significant proportion of these mice develop diabetes abruptly at 12-16 weeks of age (Hashimoto, 2011; Garcia-Barrado et al., 2011). Also, IRS2-deficient mice show central leptin resistance resulting in alterations in the control of neuropeptides in the arcuate nucleus (Masaki et al., 2012). Interestingly, the magnitude of glucose deregulation in IRS2-deficient mice can be variable, with certain congenic strains only presenting prediabetic changes (Hashimoto, 2011). This phenotypic divergence might allow an experimental tool to analyze pathogenic factors involved in central insulin signalling pathways leading to two different outcomes: prediabetes or overt diabetes (Cai, 2012). In a previous study (Burgos-Ramos et al., 2012), we found activation of hypothalamic inflammatory pathways that differentially correlate with the absence or presence of diabetes in IRS2-deficient mice, suggesting that the inflammatory process could be related to the onset of disease.

Induction of oxidative stress actively participates in tissue damage caused by hypoglycaemia and diabetes, and results in deterioration of glucose homeostasis (Singh et al., 2004). The mechanisms and pathways involved in this process are complex. If reactive oxygen species (ROS) are not detoxified, cellular components are damaged and stress-sensitive intracellular signalling pathways mediated by nuclear factor-kappa B (NFκB), p38 and Jun N-terminal kinase [JNK; also known as stress-activated kinases (SAPK)] are altered (Newsholme et al., 2007). Although the brain consumes 20% of the oxygen in the body and has a high content of unsaturated fatty acids and catecholamines that are readily oxidized, it has a low content of antioxidants (Rizzo et al., 2010), which makes it a susceptible organ to oxidative damage (Uttara et al., 2009). Oxidation produces lipid peroxides and DNA oxides that produce cell disturbances that lead to inflammation and deterioration of the central nervous system. Multiple mechanisms have been proposed to protect cells from apoptosis, including the activation of insulin signalling mediators through IRS2, such as the phosphoinositide 3-kinase (PI3K)/protein kinase B (Akt) (Franke et al., 1997; Valverde et al., 2004) and Ras/mitogen-activated protein kinases (MAPK) pathways (Schubert et al., 2004). Hence, in the present study, we analyzed whether diabetes caused by IRS2 deficiency affects cell death in the hypothalamus, because this neuroendocrine organ is a key target for inflammation and oxidative stress in diabetes (Cai and Liu, 2011), and we analyzed the mechanisms involved.

## RESULTS

### Cell death and apoptotic pathways in the hypothalamus of non-diabetic and diabetic *IRS2*^−/−^ mice

**Fig. 1.**
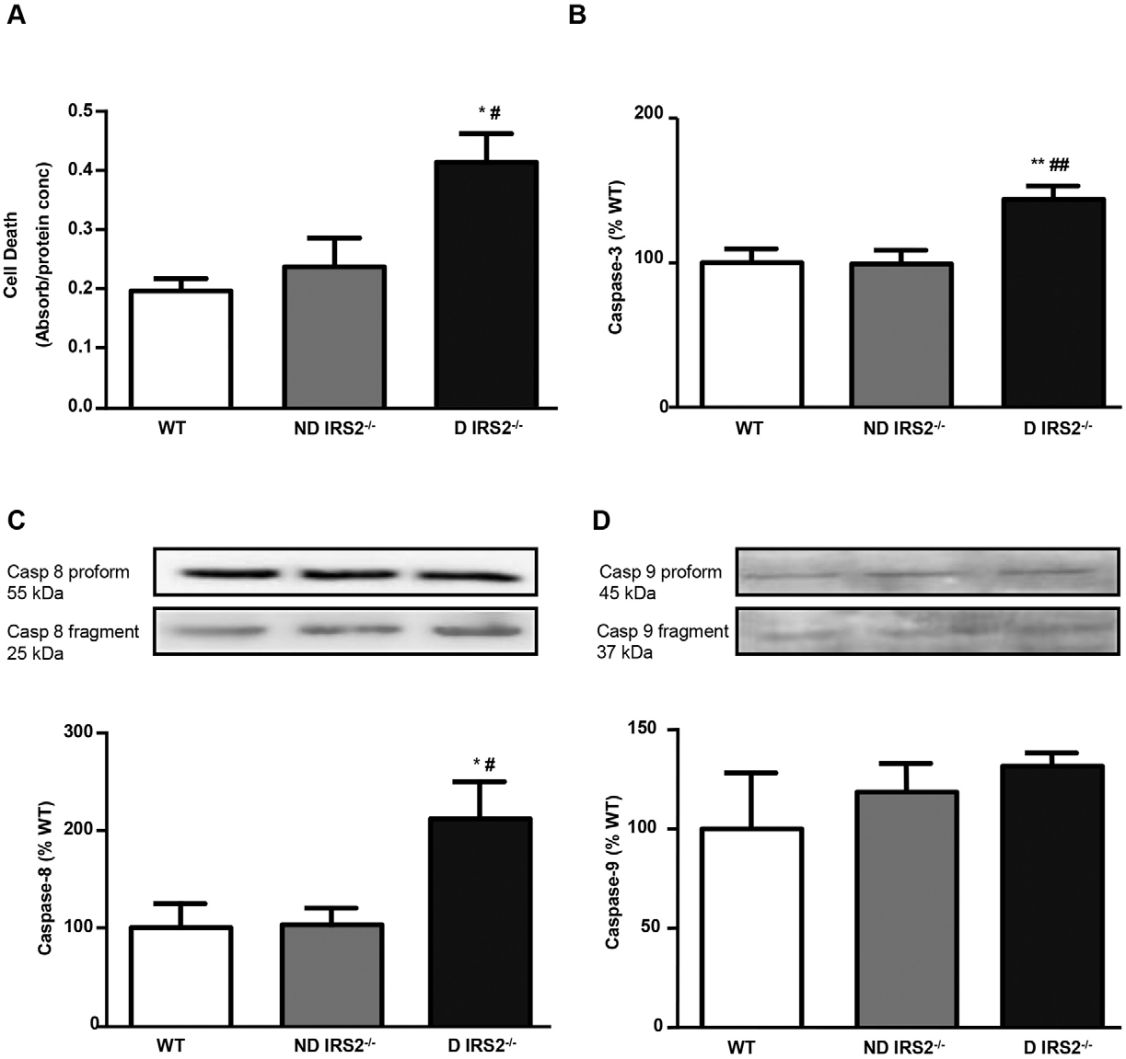
**Cell death and caspase activation in the hypothalamus of non-diabetic and diabetic *IRS2*^−/−^** **mice.** Relative levels of cell death assayed by ELISA (A), activated caspase-3 measured by bead array assay (B), caspase-8 and caspase-9 assayed by western blotting (C,D) in the hypothalamus of wild-type (WT), non-diabetic IRS2-deficient (ND IRS2^−/−^) and diabetic IRS2-deficient (D IRS2^−/−^) mice. Data are presented as means±s.e.m. Statistical significance by ANOVA: *P<0.05 and **P<0.01 vs WT mice; ^#^*P*<0.05 and ^##^*P*<0.01 vs ND *IRS2*^−/−^; *n*=6/group.

Cell death in the hypothalamus was quantified by ELISA. Only diabetic *IRS2*^−/−^ mice showed increased cell death [non-diabetic (ND): 121%; diabetic (D): 211% of wild-type (WT) values, Fig. 1A]. To determine the signalling pathways involved in the observed cell death, activation of caspases was analyzed. Activation of caspase-3, measured by bead array, was detected in diabetic *IRS2*^−/−^ mice (144% of WT values), but not in non-diabetic *IRS2*^−/−^ mice (99% of WT values, Fig. 1B). Caspase-8 (initiator caspase of the extrinsic apoptotic pathway) and caspase-9 (initiator caspase of the intrinsic pathway) were measured by western blotting. Levels of fragmented caspase-8 increased in diabetic *IRS2*^−/−^ mice (ND: 103%; D: 212% of WT values, Fig. 1C); however, levels of fragmented caspase-9 did not change in either group (Fig. 1D).

**Fig. 2.**
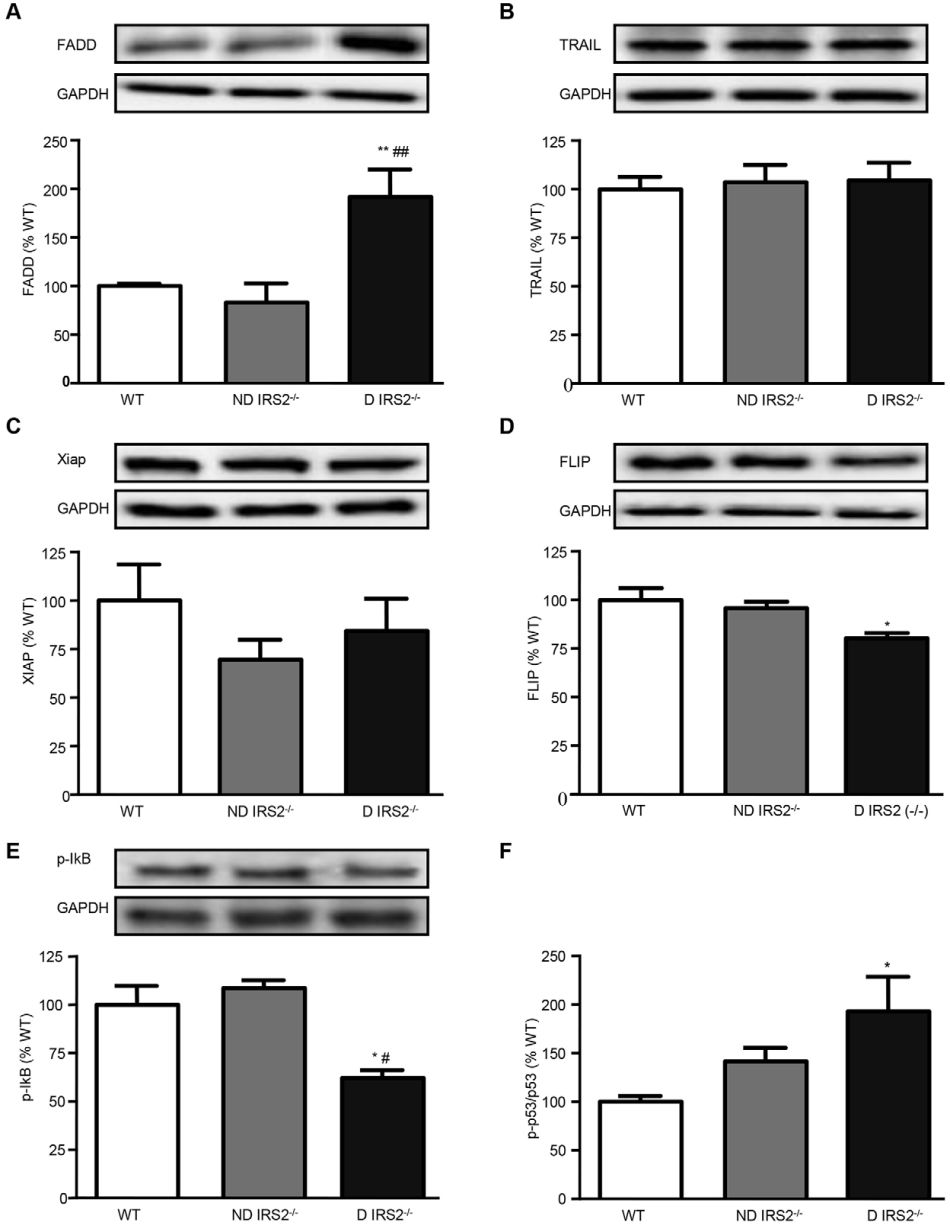
**Regulation of the extrinsic pathway of apoptosis in the hypothalamus of non-diabetic and diabetic *IRS2*^−/−^ mice.** Immunoblots probed with antibodies towards Fas-associated death domain (FADD) (A), TRAIL (B), XIAP (C), FLIP (D), phosphorylate IκB (p-IκB) (E) and phosphorylate p53 (p-p53) and p53 measured by bead array assay (F) in wild-type (WT), non-diabetic IRS2-deficient (ND IRS2^−/−^) and diabetic IRS2-deficient (D IRS2^−/−^) mice. Data are presented as means±s.e.m. Statistical significance by ANOVA: *P<0.05 and **P<0.01 vs WT mice; ^#^*P*<0.05 and ^##^*P*<0.01 vs ND *IRS2*^−/−^; *n*=6/group.

Taking into account these data, levels of TNF-related apoptosis-inducing ligand (TRAIL) and Fas-associated death domain (FADD), two proteins involved in the extrinsic pathway of apoptosis, were assayed by western blotting. Levels of FADD were increased in diabetic *IRS2*^−/−^ mice (ND: 83%; D: 192% of WT values; Fig. 2A). By contrast, levels of TRAIL were not different in diabetic and non-diabetic *IRS2*^−/−^ mice (ND: 104% and D: 105% of WT values, Fig. 2B).

Next, we studied the levels of the apoptosis inhibitory proteins X-linked inhibitor of apoptosis (XIAP) and FADD-like IL-1β-converting enzyme-inhibitory protein large (FLIP). Levels of XIAP were not statistically different among groups (ND: 70%; D: 84% of WT values, Fig. 2C), whereas diabetic *IRS2*^−/−^ mice showed decreased FLIP_L_ levels compared to the controls (ND: 96%; D: 80% of WT values, Fig. 2D).

Activation of NFκB was assayed by studying the levels of phosphorylated IκB. In diabetic *IRS2*^−/−^ mice, decreased levels of phosphorylated IκB were found (ND: 90%; D: 47% of WT value; Fig. 2E). In contrast, the mRNA levels of *Nfkbia* mRNA, which encodes IkBα, were increased in the hypothalamus of diabetic *IRS2*^−/−^ mice (ND: 117%; D 154% of WT value).

Moreover, levels of p53 increased in *IRS2*^−/−^ mice, with this increase being statistically significant only in diabetic mice (ND: 142%; D: 193% of WT mice; Fig. 2F).

**Fig. 3.**
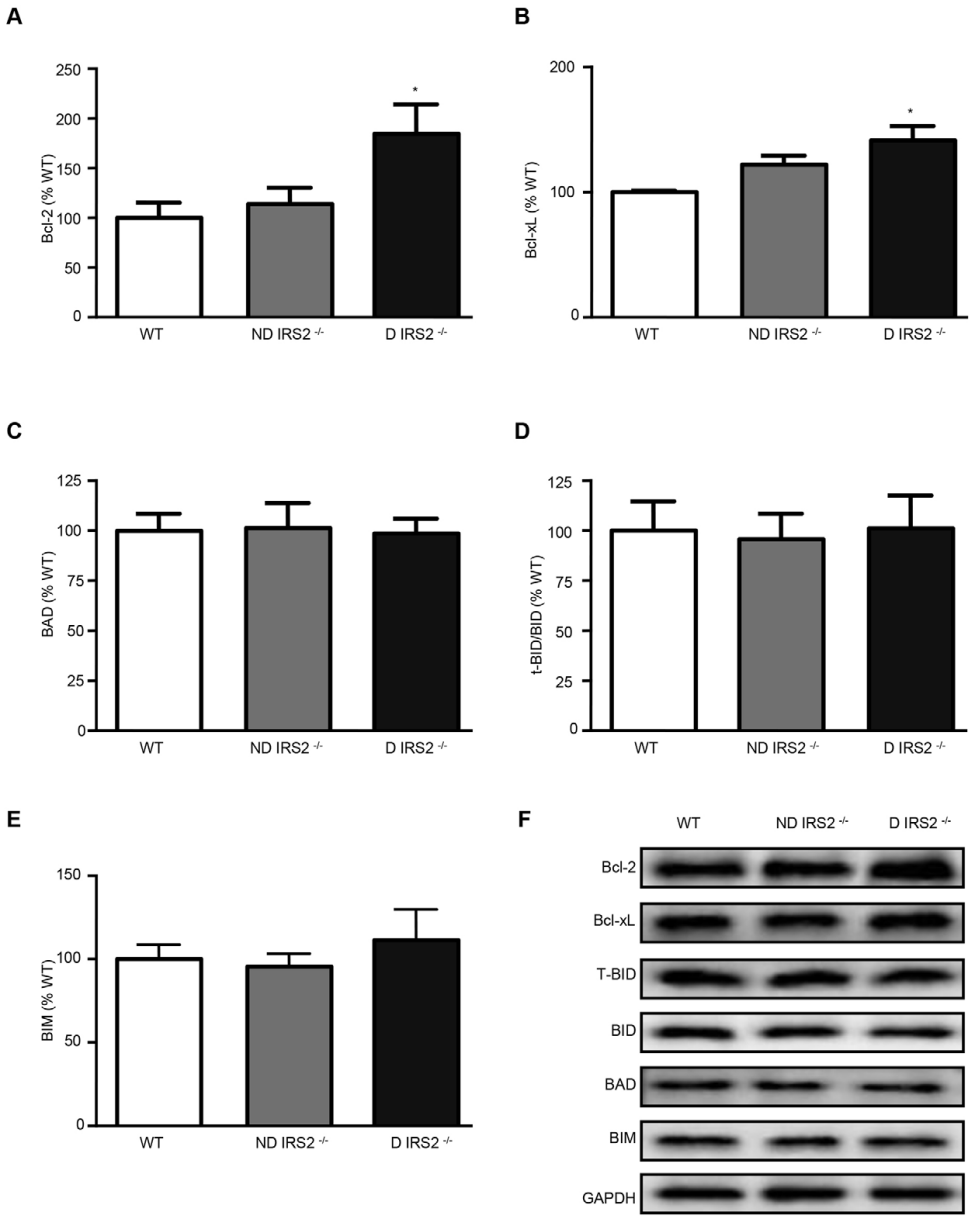
**Regulation of Bcl-2 family proteins in the hypothalamus of non-diabetic and diabetic *IRS2*^−/−^ mice.** Immunoblots probed with antibodies against Bcl-2 (A), Bcl-xL (B), BAD (C), BID (D) and BIM (E) in the hypothalamus of wild-type (WT), non-diabetic IRS2-deficient (ND IRS2^−/−^) and diabetic IRS2-deficient (D IRS2^−/−^) mice. (F) Representative immunoblotos for Bcl-2, Bcl-xL, BAD, BID, BIM and GAPDH. Data are presented as means±s.e.m. Statistical significance by ANOVA: *P<0.05 vs WT mice; *n*=6/group.

Levels of the anti-apoptotic proteins Bcl-2 and Bcl-xL, and pro-apoptotic proteins BAD, BID and BIM, were analyzed by western blotting. Bcl-2 and Bcl-xL were increased in diabetic *IRS2*^−/−^ mice (185% and 141% of WT values, respectively) but did not change in non-diabetic *IRS2*^−/−^ mice (114% and 122% of WT values, respectively; Fig. 3A,B). Levels of pro-apoptotic proteins did not change in any *IRS2*^−/−^ group regardless of diabetes: BAD (ND: 101%; D: 99% of WT values), truncated BID (t-BID)/BID (ND: 96%; D: 101% of WT values) and BIM (ND: 96%; D: 112% of WT values; Fig. 3C,D and E, respectively).

To study the inflammatory state of the hypothalamus, we measured the levels of several cytokines involved in the inflammatory process. As shown in Table 1, TNF-α, IL-6 and IL-1β were increased in the hypothalamus of diabetic *IRS2*^−/−^ mice, whereas IL-10 was decreased although the latter did not reach statistical significance. By contrast, non-diabetic *IRS2*^−/−^ mice showed reduced levels of IL-6.

**Table 1.**
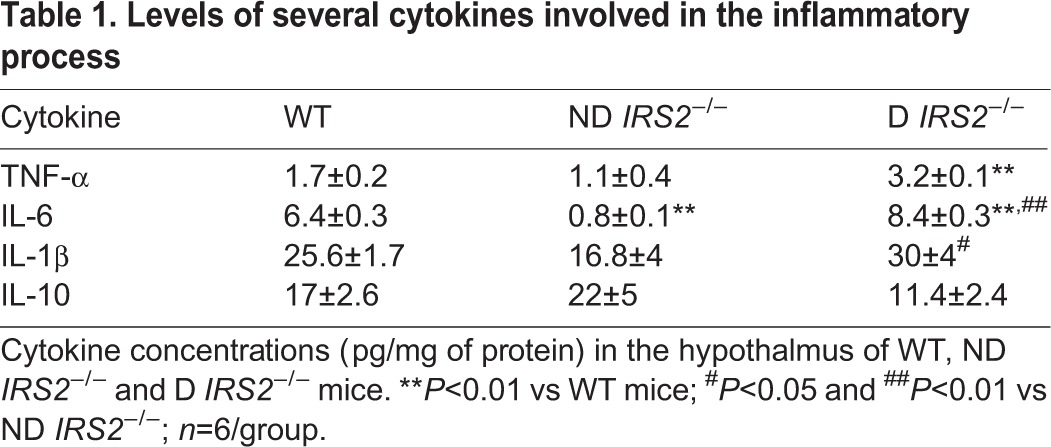
**Levels of several cytokines involved in the inflammatory process**

### Oxidative stress in the hypothalamus of non-diabetic and diabetic *IRS2*^−/−^ mice

**Fig. 4.**
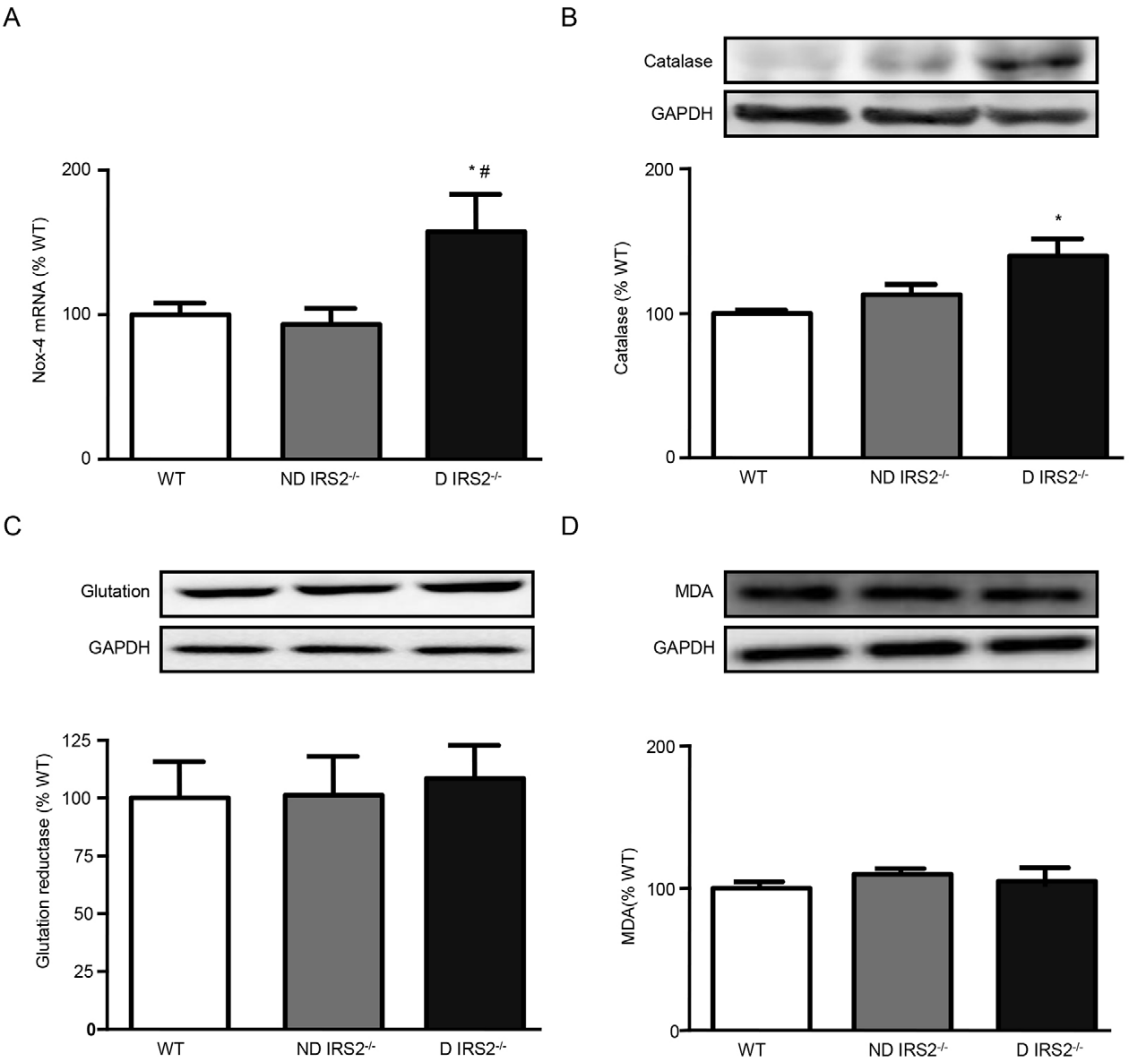
**Analysis of oxidative stress markers in the hypothalamus of non-diabetic and diabetic *IRS2*^−/−^ mice.** Relative mRNA levels of *Nox-4* (A), and immunoblots probed with antibodies against catalase (B), glutathione reductase (C) and malondialdehyde (MDA) (D) in the hypothalamus of wild-type (WT), non-diabetic IRS2-deficient (ND IRS2^−/−^) and diabetic IRS2-deficient (D IRS2^−/−^) mice. Data are presented as means±s.e.m. Statistical significance by ANOVA: *P<0.05 vs WT mice; ^#^*P*<0.05 vs ND *IRS2*^−/−^. *n*=6/group.

To study the role of oxidative stress, different enzymes were assessed. NADPH oxidase 4 (*Nox-4*) mRNA levels were increased in diabetic *IRS2*^−/−^ mice (ND: 93%; D: 157% of WT values, Fig. 4A). Catalase protein levels, measured by western blotting, were also increased in diabetic *IRS2*^−/−^ mice (ND: 113%; D: 140% of WT values; Fig. 4B).

Levels of glutathione reductase and malondialdehyde (MDA), determined by western blotting, did not change in diabetic or non-diabetic mice (glutathione reductase ND: 101%, D: 109% of WT values; MDA: ND: 102%, D: 103% of WT values; Fig. 4C).

### Identification of cell types susceptible to apoptosis in the hypothalamus of *IRS2*^−/−^ mice

**Fig. 5.**
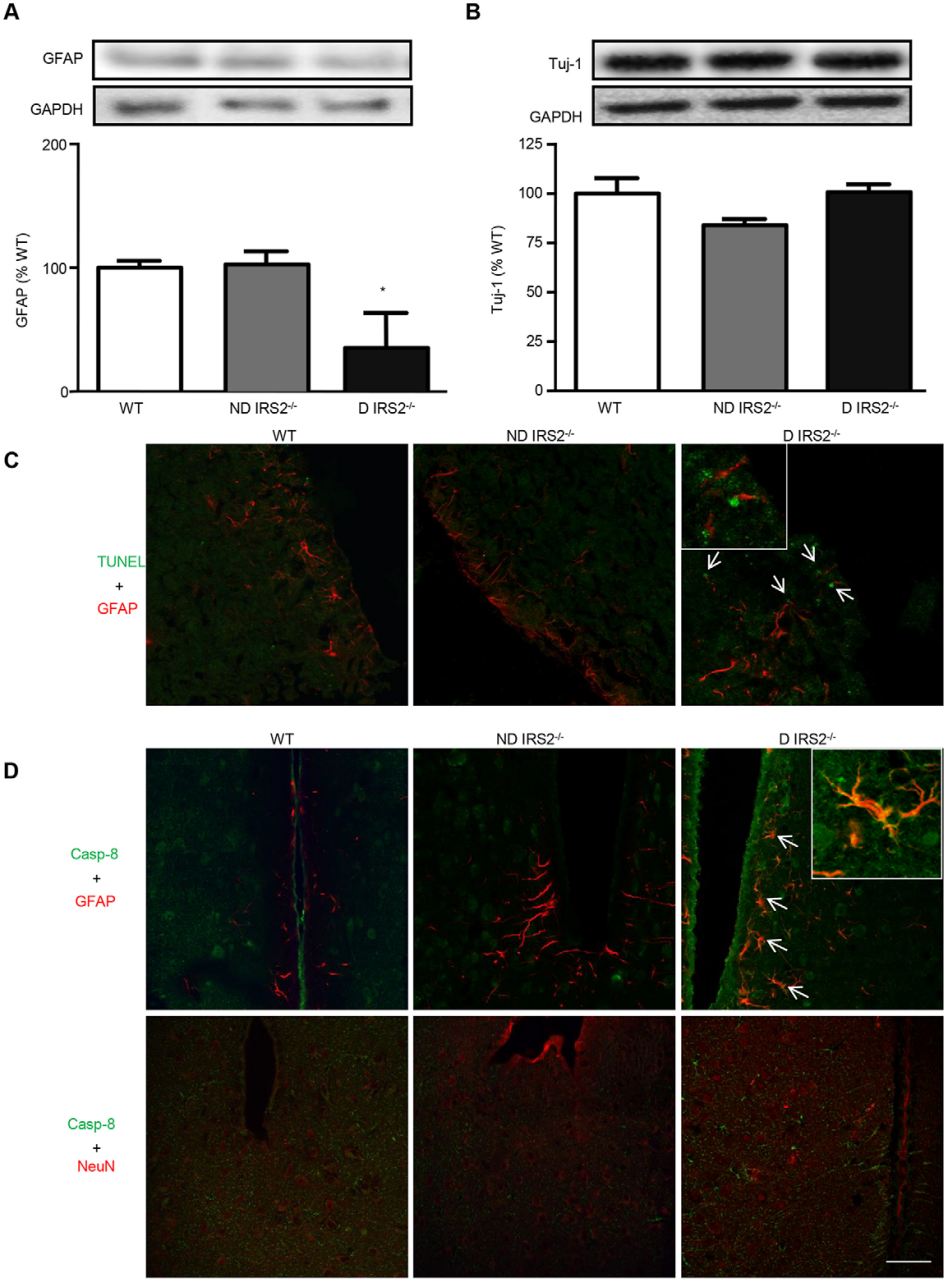
**Analysis of the cell type susceptible to apoptotic cell death in the hypothalamus of *IRS2*^−/−^ mice.** Immunoblots probed with antibodies against GFAP (A) and Tuj-1 (B) in the hypothalamus of wild-type (WT), non-diabetic IRS2-deficient (ND IRS2^−/−^) and diabetic IRS2-deficient (D IRS2^−/−^) mice. The average of three independent assays performed in duplicate is shown. Statistical significance by ANOVA: *P<0.05 vs WT mice. *n*=6/group. (C) Colocalization of TUNEL and GFAP in the hypothalamus of WT, ND *IRS2*^−/−^ and D *IRS2*^−/−^ mice. Arrows indicate astrocytes with apoptotic nuclei. (D) Colocalization of cleaved caspase-8 and GFAP, and of cleaved caspase-8 and NeuN, in the hypothalamus of WT, ND *IRS2*^−/−^ and D *IRS2*^−/−^ mice. Arrows indicate colocalization of GFAP and cleaved caspase-8. Scale bar: 50 µm; inset, 100 µm.

We measured glial fibrillary acidic protein (GFAP) as a marker of astrocytes and neuronal β-III tubulin (Tuj-1), as a marker of neurons, by western blotting. Only diabetic *IRS2*^−/−^ mice presented decreased levels of GFAP (ND: 90%; D: 26% vs WT mice). Levels of Tuj-1 were similar in all groups (ND: 84%; D: 101% vs WT mice; Fig. 5A and B, respectively).

To determine whether astrocytes are the cells dying by apoptosis in the hypothalamus of diabetic *IRS2*^−/−^ mice, we performed TUNEL assays in combination with immunofluorescence for GFAP in cryostat brain sections. As shown in Fig. 5C, TUNEL signal was increased in the hypothalamus of diabetic *IRS2*^−/−^ mice. Moreover, colocalization of TUNEL with GFAP was also observed in the hypothalamus of diabetic *IRS2*^−/−^ mice.

### Analysis of cleaved caspase-8 localization in the hypothalamus of non-diabetic and diabetic *IRS2*^−/−^ mice

To establish the type of hypothalamic cell in which caspase-8 is fragmented, brain slices were immunoassayed for cleaved caspase-8 in combination with GFAP or NeuN (a neuronal-specific antibody). Inmunostaining for cleaved caspase-8 was scarce in the hypothalamus of WT and non-diabetic *IRS2*^−/−^ mice. By contrast, cleaved caspase-8 expression increased in diabetic *IRS2*^−/−^ mice and colocalized mainly with GFAP (Fig. 5D).

### Analysis of IGF-I and IGF-IR levels in the hypothalamus of non-diabetic and diabetic *IRS2*^−/−^ mice

**Fig. 6.**
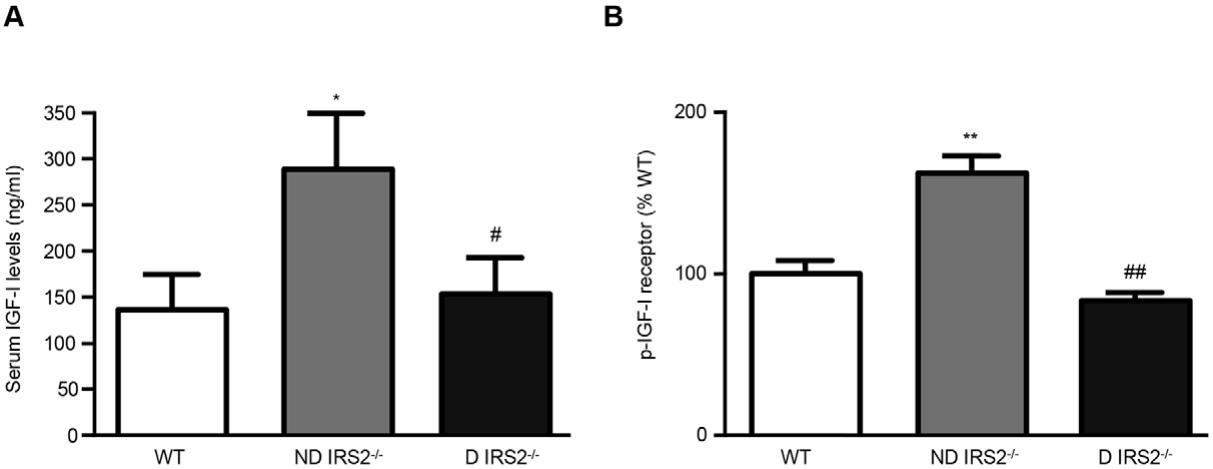
**IGF-I and IGF-IR levels.** Serum levels of IGF-I assayed by ELISA (A) and relative levels of phosphorylated IGF-IR in the hypothalamus measured by bead array assay (B) of wild-type (WT), non-diabetic IRS2-deficient (ND IRS2^−/−^) and diabetic IRS2-deficient (D IRS2^−/−^) mice. Data are presented as means±s.e.m. Statistical significance by ANOVA: *P<0.05 and **P<0.01 vs WT mice; ^#^*P*<0.05 and ^##^*P*<0.01 vs ND IRS2^−/−^; *n*=6/group.

We measured serum IGF-I levels by ELISA and observed an increase in non-diabetic *IRS2*^−/−^ mice as compared to the WT controls (WT: 140 ng/ml; ND: 295 ng/ml; D: 153 ng/ml; Fig. 6A). Moreover, the hypothalamic levels of phosphorylated IGF-IR were increased in non-diabetic *IRS2*^−/−^ mice (162% of WT), but *IRS2*^−/−^ diabetic mice showed levels of IGF-IR that were not different from those of WT mice (83%; Fig. 6B).

## DISCUSSION

The hyperglycaemia associated with diabetes causes cellular dysfunction through various mechanisms and this can result in tissue injury. Diabetic *IRS2*^−/−^ mice are hyperglycaemic (Withers et al., 1998, 1999; González-Rodríguez et al., 2010; Burgos-Ramos et al., 2012) and hyperglycaemia itself activates NADPH oxidases to accelerate oxidative stress (Koike et al., 2007), which is considered an important mediator of diabetic complications (Inoguchi et al., 2007). In order to study the role of oxidative stress and the relationship with inflammatory processes described in diabetes, we analyzed the levels of key enzymes involved in oxidative stress in the hypothalamus of *IRS2*^−/−^ mice and found that protein levels of catalase were increased in diabetic *IRS2*^−/−^ mice. This effect could be attributed to higher levels of H_2_O_2_, reflected by increased *Nox-4* mRNA levels, because H_2_O_2_ is the major form of ROS generated by this NADPH oxidase (Lambeth, 2007; Serrander et al., 2007).

Oxidative stress induced by hyperglycaemia serves as a key trigger of inflammatory gene expression (Gumieniczek et al., 2005). Pro-inflammatory cytokines play an important role in the pathology of diabetes (Bodles and Barger, 2004). TNF-α has been suggested to participate in the development of diabetes by impairing insulin actions (Lechleitner et al., 2000; Tuttle et al., 2004). TNF-α also stimulates the production of ROS that directly signal cells to undergo apoptosis (Kim et al., 2010). In this regard, we found increased levels of cell death in the hypothalamus of diabetic *IRS2*^−/−^ mice, compared with non-diabetic *IRS2*^−/−^ and WT mice, that could be due to increased levels of pro-inflammatory cytokines such as TNF-α, IL-6 and IL-1β. TNF-α could activate caspase-8, involving the adaptor protein FADD (MORT1); this activation is essential to bridge members of the TNF receptor (TNFR) superfamily to procaspase-8 to assemble the death-inducing signalling complex (DISC) during apoptosis (Micheau and Tschopp, 2003). In turn, caspase-8 can then activate caspase-3, although a direct pathway for caspase-8-elicited apoptosis has also been described (Benchoua et al., 2002). The intrinsic and extrinsic pathways are not completely independent. In fact, in some cells, activation of caspase-8 results in the activation of the mitochondrial pathway through the cleavage of the BH3-only proteins BIM, BID and p53 up-regulated modulator of apoptosis (PUMA); this is essential for the activation of the BAX- and BAK-dependent cell death program (Ren et al., 2010; Favaloro et al., 2012). However, our results showed that BIM and BID were not cleaved in the hypothalamus of *IRS2*^−/−^ mice, therefore excluding the involvement of the intrinsic pathway in apoptosis. These results were also supported by the lack of activation of caspase-9.

Other relevant proteins involved in apoptosis that have been found to be upregulated in the hypothalamus of diabetic *IRS2*^−/−^ mice are the transcription factor p53 and NFκB. p53, a key factor in apoptosis (Reinhardt and Schumacher, 2012), is activated in response to oxidative stress and DNA damage induced by H_2_O_2_ (Han et al., 2008). Furthermore, the activation of caspase-8 has been implicated in p53-mediated apoptosis (Haupt et al., 2003). Hyperglycaemia has also been shown to activate p53, resulting in cell death (Eid et al., 2010). In addition, TNF-α, which was elevated in the serum and hypothalamus of diabetic *IRS2*^−/−^ mice together with IL-6 and IL-1β, could activate both caspase-8 and p53 in the hypothalamus of diabetic *IRS2*^−/−^ mice, and this activation could explain the elevated cell death. On the other hand, NFκB has been associated with the extrinsic cell-death pathway because it can be activated by pro-apoptotic signals, including death receptors (Dickens et al., 2012). However, NFκB has a dual role and can also participate in cell survival (Morgan and Liu, 2011; Singh et al., 2015) through the activation of inhibitors of apoptosis (IAPs) and other survival proteins such as Bcl-2 and Bcl-xL (Dolcet et al., 2005). Although there are a few exceptions, NFκB contributes to cell death (Perkins and Gilmore, 2006) in most cases. In this regard, various recent reports have shown that overexpression of NFκB impairs survival, proliferation and differentiation of hypothalamic neural stem cells, with an increase in apoptosis by induction of pro-apoptotic members of Bcl-2 family and caspases and upregulation of anti-apoptotic genes (Li et al., 2012). Therefore, the increased levels of NFκB might be associated with activation of the extrinsic cell-death pathway and contribute to the cell death found in the hypothalamus of diabetic *IRS2*^−/−^ mice, although further studies directed to inhibit inflammatory mediators are necessary to complete this issue. Moreover, the increase in pro-inflammatory cytokines in the hypothalamus could lead to a decay of *Nfkbia* mRNA levels, which encodes IkBα. The resulting loss of IkBα could trigger the activation of hypothalamic NFκB, which in turn might be responsible for the inflammatory changes (Yan et al., 2014). However, we found increased *Nfkbia* mRNA levels in diabetic *IRS2*^−/−^ mice, with overexpression of p65-NFκB that could increase the transcription of *Nfkbia* mRNA in an auto-regulatory loop, ensuring that NFκB is retained in the cytoplasm until cells are specifically induced to translocate it to the nucleus (Scott et al., 1993). Our results suggest that the IkBα feedback could be dependent on NFκB but other processes dependent on the molecular characteristics of the protein itself are probably involved; for example, import, export and modulation of half-life (Fagerlund et al., 2016).

One of the main signalling pathways that intersects with NFκB with regards to ROS and cell death is the crosstalk that occurs between NFκB and JNK (Morgan et al., 2008). As previously reported (Burgos-Ramos et al., 2012), levels of JNK are higher in the hypothalamus of diabetic *IRS2*^−/−^ mice and phosphorylated IκB is downregulated. Furthermore, NFκB and p53 transcriptionally regulate the *c-FLIP* gene. A decrease in c-FLIP_L_ was observed in the hypothalamus of diabetic *IRS2*^−/−^ mice. The anti-apoptotic protein c-FLIP contains a death effector domain (DED) and suppresses caspase-8 activation, preventing apoptotic processes (Safa, 2012). Whereas NFκB suppresses c-FLIP transcription (Safa et al., 2008; Bagnoli et al., 2010), p53 might induce *c-FLIP* gene transcription and c-FLIP degradation (Safa and Pollok, 2011). c-FLIP_L_ possesses dual functions: at high levels it can inhibit the activation of caspase-8 induced by Fas, but at low levels it enhances caspase-8 activation (Safa et al., 2008; Bagnoli et al., 2010) as observed in the hypothalamus of diabetic *IRS2*^−/−^ mice. On the other hand, H_2_O_2_, which is the most diffusible ROS, decreases c-FLIP expression in a dose-dependent manner (Nitobe et al., 2003). Based on this, downregulation of c-FLIP could be one possible mechanism by which the hypothalamus of diabetic *IRS2*^−/−^ mice is prone to apoptosis.

To determine which population of cells is dying in the hypothalamus of diabetic *IRS2*^−/−^ mice, we measured GFAP and Tuj-1 as markers of astrocytes and neurons, respectively. We found that only diabetic *IRS2*^−/−^ mice showed decreased levels of GFAP. On the other hand, levels of Tuj-1 were similar in all groups. TUNEL assays in combination with immunofluorescence for GFAP confirmed that astrocytes were dying by apoptosis in the hypothalamus of diabetic *IRS2*^−/−^ mice. Likewise, activated caspase-8 was also colocalized mainly with GFAP in the hypothalamus of diabetic *IRS2*^−/−^ mice. Previous studies in rats have demonstrated that caspase-8 is present in the brain, particularly in neurons and astrocytes, and plays a key role in the apoptotic cell death response after injury (Villapol et al., 2007). Of note, because cell death is a continuous process, cells can be found in different stages. Astrocytes with active caspase-8 are probably at an early process of apoptosis.

Insulin-like peptides are neuroprotective and might be involved in the pathophysiology of a number of neurological diseases, representing possible therapeutic objectives for these disorders (Benarroch, 2012; Werner and LeRoith, 2014). In particular, IGF-I exerts anti-apoptotic/pro-survival actions in a variety of systems, including the brain (Guan et al., 2015). Furthermore, IGF-I is a potent neuroprotective agent and also protects against oxidative stress induced by the lipid-peroxidizing agent H_2_O_2_ (Heck et al., 1999). The activation of the insulin receptor or IGF-IR transduces their biological effects by tyrosine phosphorylation of IRSs and Src homology 2 domain containing (Shc), and this, in turn, initiates a signalling cascade through the PI3K/Akt and Ras/MAPK kinase pathways (Siddle, 2011). Additionally, IGF-I mediates the neuroprotection exerted by some substances by augmenting the levels of anti-apoptotic proteins such as Bcl-2 and decreasing the levels of the pro-apoptotic protein BAX and the activation of caspases (Jiang et al., 2015). Non-diabetic *IRS2*^−/−^ mice did not manifest an increase in cell death in the hypothalamus, which could possibly be due to the increased levels of circulating IGF-I and the enhanced phosphorylation of the IGF-IR that would lead to the activation of survival pathways through ERK and Akt as previously reported (Burgos-Ramos et al., 2011), although further studies would be necessary to clearly demonstrate this issue.

**Fig. 7.**
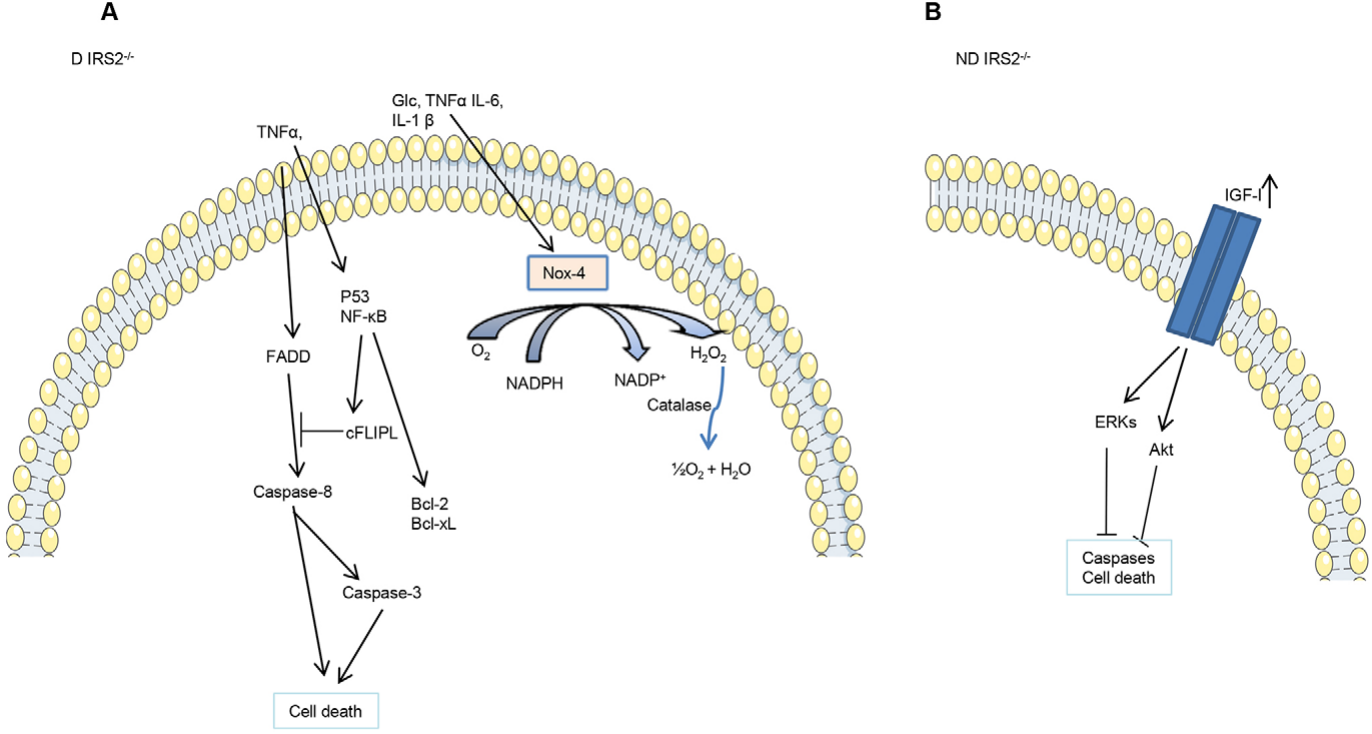
**Graphical summary.** Diagram representing the mechanism proposed for activation of cell death in astrocytes of diabetic *IRS2*^−/−^ (D IRS2^−/−^) mice in the hypothalamus (A) or inhibition of cell death in the hypothalamic astrocytes of non-diabetic *IRS2*^−/−^ (ND IRS2^−/−^) mice in the hypothalamus (B).

In summary, our findings suggest that the diabetic condition in *IRS2*^−/−^ mice entails an increase in oxidative stress and inflammation in the hypothalamus that finally could result in cell death via activation of the extrinsic apoptotic pathway. By contrast, non-diabetic *IRS2*^−/−^ mice did not show increased hypothalamic cell death, possibly owing to the rise in circulating IGF-I levels and IGF-IR phosphorylation, which would lead to stimulation of survival pathways (summarized in Fig. 7). Hence, the exposure to an inflammatory and oxidative milieu results in a diabetic-induced hypothalamic injury in this model.

## MATERIALS AND METHODS

### Materials

Electrophoresis reagents were from Bio-Rad Laboratories (Hercules, CA) and the rest of the chemicals and reagents were purchased from Sigma or Merck (Barcelona, Spain) unless otherwise indicated.

### Animals

All procedures were carried out in accordance with the local ethics committee and complied with Royal Decree 53/2013 pertaining to the protection of experimental animals and with the European Communities Council Directive (2010/63/EU).

Wild-type (WT) and *IRS2*^−/−^ mice, maintained on a similar mixed genetic background (C57BL/6×129sv), were purchased from the Jackson Laboratory (Bar Harbor, ME). Adult (11- to 12-week-old) male mice were housed individually and maintained in a ventilated room at a constant temperature (22°C) and humidity (50%) with free access to standard chow and water on a 12-h light/dark cycle. In this study, we grouped mice in: WT mice as controls, diabetic *IRS2*^−/−^ (D *IRS2*^−/−^) with non-fasting glucose levels over 500 mg/dl (determined by the glucose oxidase method by using the Precision G glucose meter; Abbott Laboratories, North Chicago, IL), and age-matched non-diabetic *IRS2*^−/−^ mice (ND *IRS2*^−/−^) with glucose levels under 200 mg/dl (*n*=6 in each group). At 6-7 days after the debut of diabetes, animals were sacrificed by decapitation at 10:00 a.m. under non-fasting conditions. Mice from the control and ND *IRS2*^−/−^ groups were sacrificed at the same time as the D *IRS2*^−/−^ mice. Trunk blood was collected in cooled tubes and centrifuged at 3000 ***g*** for 10 min at 4°C. The serum was stored at −80°C until processed.

### Protein extraction and quantification

The hypothalami were homogenized on ice in 250 μl of lysis buffer (pH 7.6) containing EDTA (10 mM), HEPES 50 (mM), sodium pyrophosphate (50 mM), NaF (0.1 M), Na_3_VO_4_ (10 mM), 1% Triton X-100, phenylmethylsulfonyl fluoride (2 mM), leupeptin (10 µg/ml) and aprotinin (10 µg/ml). The lysates were incubated overnight at −80°C and then clarified by centrifugation at 12,000 ***g*** for 5 min at 4°C. The supernatants were transferred to fresh tubes and stored at −80°C until assayed. Total protein concentration was determined by the method of Bradford (Bio-Rad).

### Western blot

A total of 40 µg of protein was loaded in all wells and resolved using an 8-12% SDS-PAGE and then transferred onto polyvinyl difluoride (PVDF) membranes (Bio-Rad). Filters were blocked with Tris-buffered saline containing 0.1% Tween 20 (TTBS) with 5% (w/v) bovine serum albumin (BSA) or non-fat milk during 2 h at 25°C and incubated overnight at 4°C with the primary antibody at a dilution of 1:1000 in blocking buffer. Primary antibodies included: phosphorylated IκB from Cell Signaling Technology (Danvers, MA); BAD and glutathione reductase from Santa Cruz Biotechnology Inc. (Santa Cruz, CA); anti-FADD (M033-3; Clone 1F7) and caspase-9 from MBL International (Woburn, MA); caspase-8 from Neomarkers (Fremont, CA); TRAIL, Bcl-2, BID and β-III tubulin (Tuj-1) from R&D Systems (Minneapolis, MN); XIAP and Bcl-xL from BD Transduction Laboratories (Franklin Lakes, NJ); FLIP, catalase and GFAP from Sigma-Aldrich (St Louis, MO), BIM from BD Pharmingen (Mississauga, ON, Canada), and malondialdehyde from Cell Biolabs (San Diego, CA). The membranes were washed three times with TTBS and incubated with the corresponding secondary antibody conjugated with peroxidase (Thermo Fisher Scientific Inc., Waltham, MA) at a dilution of 1:2000 in non-fat milk during 90 min at 25°C. The proteins were detected by chemiluminescence using an immune-star western chemiluminiscent kit (Bio-Rad) and quantified by densitometry using a Kodak Gel Logic 1500 Image Analysis system and Molecular Imaging Software version 4.0 (Rochester, NY). All blots were re-blotted with anti-glyceraldehyde-3-phosphate dehydrogenase (GAPDH, AnaSpec, San Jose, CA) to normalize for gel-loading variability.

### Bead array assay

The content of TNF-α, IL-6, IL-1β, IL-10 and phosphorylated and total p53 and IGF-IR were measured by using a bead array assay (Merck Millipore, Darmstadt, Germany) as previously described (Khan et al., 2004). Briefly, beads conjugated to antibody and lysates (50 μl each) were incubated for 18 h at 25°C, washed and incubated with biotin-conjugated antibody (25 μl) for 30 min. Then the beads were incubated with 50 μl streptavidin conjugated to phycoerythrin (PE) (streptavidin-PE, diluted 1:100) for 30 min. Fluorescence was analyzed using a Bio-Plex suspension array system 200 (Bio-Rad Laboratories). Raw data [mean fluorescence intensity (MFI)] were analyzed using the Bio-Plex Manager software 4.1 (Bio-Rad Laboratories). For caspase-3 determination, 100 μg of protein and beads were incubated at 700 rpm for 2 h at 25°C, washed and posterior incubations performed for 1 h.

### RNA purification

Total RNA was extracted following the instructions of TriReagent (Invitrogen, Carlsbad, CA). Briefly, each hypothalamus was homogenized in 1 ml of TriReagent and incubated for 5 min at room temperature (RT) to dissociate nucleoprotein complexes. Chloroform (0.2 ml) was added and samples were vortexed, incubated for 15 min at RT and then centrifuged at 12,000 ***g*** for 15 min at 4°C. The aqueous phase was transferred to new tubes and isopropanol (0.5 ml) was added to precipitate RNA. Samples were incubated for 10 min at RT and then centrifuged at 12,000 ***g*** for 10 min at 4°C. Pellets were washed in 75% ethanol (1 ml), centrifuged at 7500 ***g*** for 5 min at 4°C, and dissolved in RNase-free water. Absorbance at 260 nm was measured to determine concentrations.

### Reverse transcription (RT) and real-time RT-PCR

The reverse transcription reaction was performed on 2 μg of total RNA using the high-capacity cDNA kit (Applied Biosystems, Foster City, CA). Real-time RT-PCR was performed in an ABI Prism 7000 Sequence Detection System (Applied Biosystems). *Nfkbia* mRNA levels were measured using the TaqMan gene expression assay (Mm00477798_m1; Applied Biosystems). The PCR mixture contained 300 nM of each primer. Relative gene expression comparisons were carried out using an invariant endogenous control (GAPDH). *Nox-4* mRNA levels were measured with SYBR Green (Roche, Mannheim, Germany), with primers purchased from Sigma. The forward and reverse sequences were the following: 5′-TCCAAGCTCATTTCCCACAG-3′ and 5′-CGGAGTTCCATTACATCAGAGG-3. The PCR mixture contained 300 nM of each primer. Relative gene expression comparisons were carried out using an invariant endogenous control (GAPDH; 4352339E). According to the manufacturer's guidelines, the ΔΔC_T_ method was used for relative quantification.

### Immunoenzymometric assay (IEMA) for determination of IGF-I in serum

The quantitative determination of serum IGF-I was performed with the OCTEIA immunoenzymometric assay from IDS, Immunodiagnostic Systems Limited (Boldon, Tyne and Wear, UK). The method was performed according to the manufacturer's instructions. Briefly, serum samples were incubated with a reagent to inactivate binding proteins (10 min) and then diluted for assay. Samples were added to antibody-coated wells for 2 h, at RT on a shaking platform. The wells were washed and horseradish peroxidase was added (30 min, RT); after washing, a solution of tetra-methyl-benzidine was added to develop colour (30 min, RT). The reaction was stopped and the absorbance read (450 nm; reference 650 nm) in a microtiter plate reader (Tecan Infinite M200, Grödig, Austria), with colour intensity being directly proportional to the amount of rat IGF-I present in the sample. This assay has a sensitivity limit of 63 ng/ml. The intra- and inter-assay coefficients of variation were 4.3% and 6.3%, respectively.

### Cell death detection ELISA

This assay was carried out according to the manufacturer's instructions (Roche). Briefly, tissue was homogenized in incubation buffer and microtiter plates were coated with anti-histone antibody. The samples were added (in duplicate) and incubated (90 min, RT). The wells were then washed and incubated with anti-DNA-peroxidase (90 min, RT). After washing, substrate solution was added until the colour developed adequately (approximately 15 min). The samples were measured at 405 nm on an automatic microplate analyzer (Tecan Infinite M200, Grödig, Austria). Background measurements at 490 nm were made and this value subtracted from the mean value of each sample.

### TUNEL plus immunohistochemistry

Cell death detection by TUNEL assay was performed following the manufacturer's instructions (Roche). Brieﬂy, after ﬁxation in 4% paraformaldehyde in 0.1 M phosphate buffer (pH 7.4), cryostat brain sections (20 µM) were washed three times in phosphate buffer and incubated for 30 min with a 0.1% sodium citrate, 0.1% Triton X-100 solution to increase tissue permeability. Slides were again washed, and incubated with TUNEL solution for 90 min at 37°C in a humid chamber in the dark. After washing, the slides were incubated with an anti-GFAP antibody (1:2000), in TBS containing 3% BSA and 1% Triton X-100 and left for 48 h at 4°C. The slides were incubated with Alexa Fluor anti-ﬂuorescein-488 and -633-conjugated goat anti-mouse IgG (1:2000; Molecular Probes, Eugene, OR) in blocking buffer, both at a dilution of 1:1000. Finally, after washing, the slides were mounted in Clear Mount (Electronic Microscopy Sciences, Hartfield, PA). Immunofluorescence was visualized directly by using a DM IRB confocal microscope (Leica, Wetzlar, Germany).

### Immunofluorescence

Double-immunofluorescence for GFAP and active caspase-8 (Novus Biologicals Europe, Cambridge, UK) or NeuN (Merck Millipore) and active caspase-8 were carried out on sections (30 µm) obtained on a vibratome. Sections were blocked in phosphate buffer 0.1 M pH 7.4 containing 3% BSA, 1% Triton X-100 for 24 h at 4°C. Afterwards, sections were incubated with anti-GFAP (1:500) or anti-NeuN (1:500) and anti-active capase-8 (1:500) diluted in blocking buffer for 48 h at 4°C. Sections were then washed with phosphate buffer (PB) with 0.1% Triton X-100 and incubated for 90 min at RT with anti-rabbit IgG-biotin (1:1000; Thermo Scientific) diluted in blocking buffer, washed and incubated with streptavidin Alexa Fluor 488 (1:1000, Molecular Probes) and Alexa Fluor 633 anti-mouse IgG (1:1000) for 90 min at RT. After washing, sections were cover-slipped with Clear Mount. Immunofluorescence was visualized directly by using a DM IRB confocal microscope.

### Statistical analysis

All results are presented as mean±s.e.m. Statistical analysis of all data was carried out by one-way ANOVA followed by a Bonferroni's test. The values were considered significantly different when the *P-*value was less than 0.05. Statistical analyses were performed using Prisma software 4.0 (Prisma, GraphPad, San Diego, CA).
